# Supplementary material for: PLCβ2 negatively regulates the inflammatory response to virus infection by inhibiting phosphoinositide-mediated activation of TAK1
Source: Nat Commun. 2019 Feb 14;10:746. doi: 10.1038/s41467-019-08524-3 (PMC6375925; doi:10.1038/s41467-019-08524-3)
Supplement: Supplementary file 1 — Supplementary Information [file 41467_2019_8524_MOESM1_ESM.pdf]

PLCβ2 Negatively Regulates the Inflammatory Response to Virus Infection by Inhibiting Phosphoinositide-mediated Activation of TAK1

Wang et al.

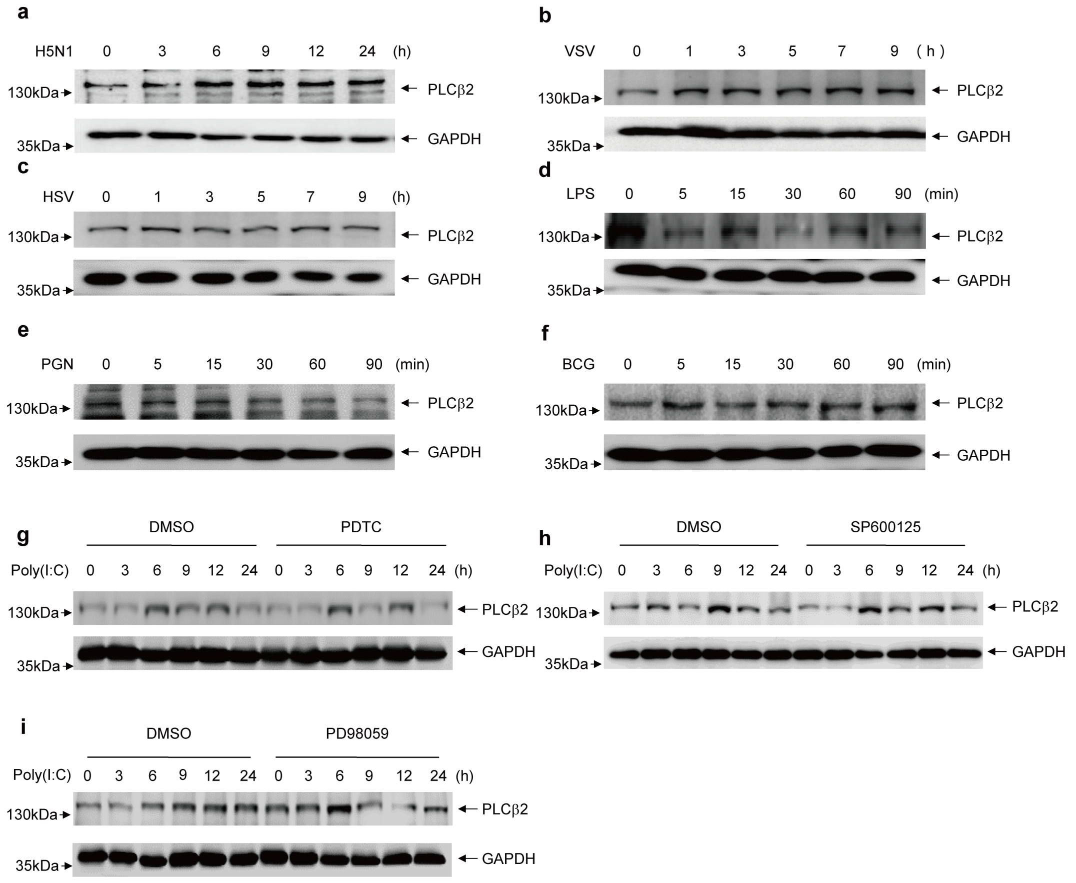

Supplementary Figure 1 RNA virus infection specifically induces PLCβ2 expression (**a**-**f**) IB of PLCβ2 in lysates of mouse peritoneal macrophages infected or stimulated with H5N1 (**a**), VSV (**b**), HSV (**c**), LPS (**d**), PGN (**e**) or BCG (**f**) for indicated times. (**g**-**i**) IB of PLCβ2 in mouse peritoneal macrophages pretreated for 1 h with the NF-κB inhibitor PDTC (**g**), JNK inhibitor SP600125 (**h**) or MEK inhibitor PD98059 (**i**) before poly(I:C) stimulation for the indicated times. Data are representative of three experiments with at least three independent biological replicates (mean and s.e.m. of n = 3 cultures in **a**-**i**).

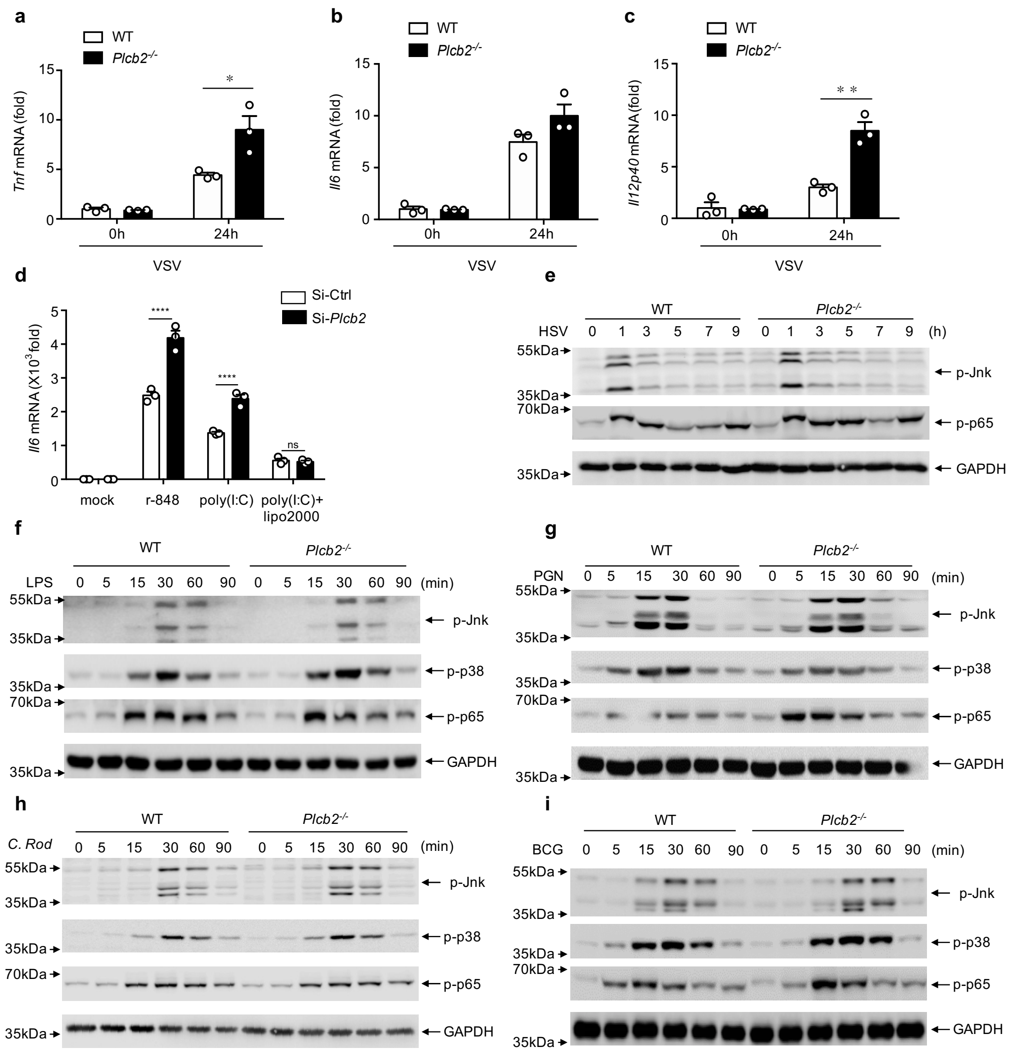

Supplementary Figure 2 PLCβ2 suppresses RNA virus- induced inflammation (**a-c**) Q-PCR analysis of relative *Tnf* (**a**)*, Il6* (**b**) and *Il12p40* (**c**) mRNA in wild-type or *Plcb2*$^{-/-}$ macrophages infected with VSV for the indicated times. (**d**) Q-PCR analysis of relative *Il6* in *Plcb2* knockdown macrophages stimulated with r-848 (TLR7 ligand) or poly(I:C) for 4 hours or transfected with poly(I:C) for 6 hours. **(e-i)** IB of lysates from wild-type or *Plcb2*$^{-/-}$ macrophages infected or stimulated with HSV (**e**), LPS (**f**), PGN (**g**), C.Rod (**h**) or BCG (**i**) for the indicated times using phospho-antibodies. Data are representative of three experiments with at least three independent biological replicates (mean and s.e.m. of n=3 mice in **a** or n = 3 cultures in **d-i**).

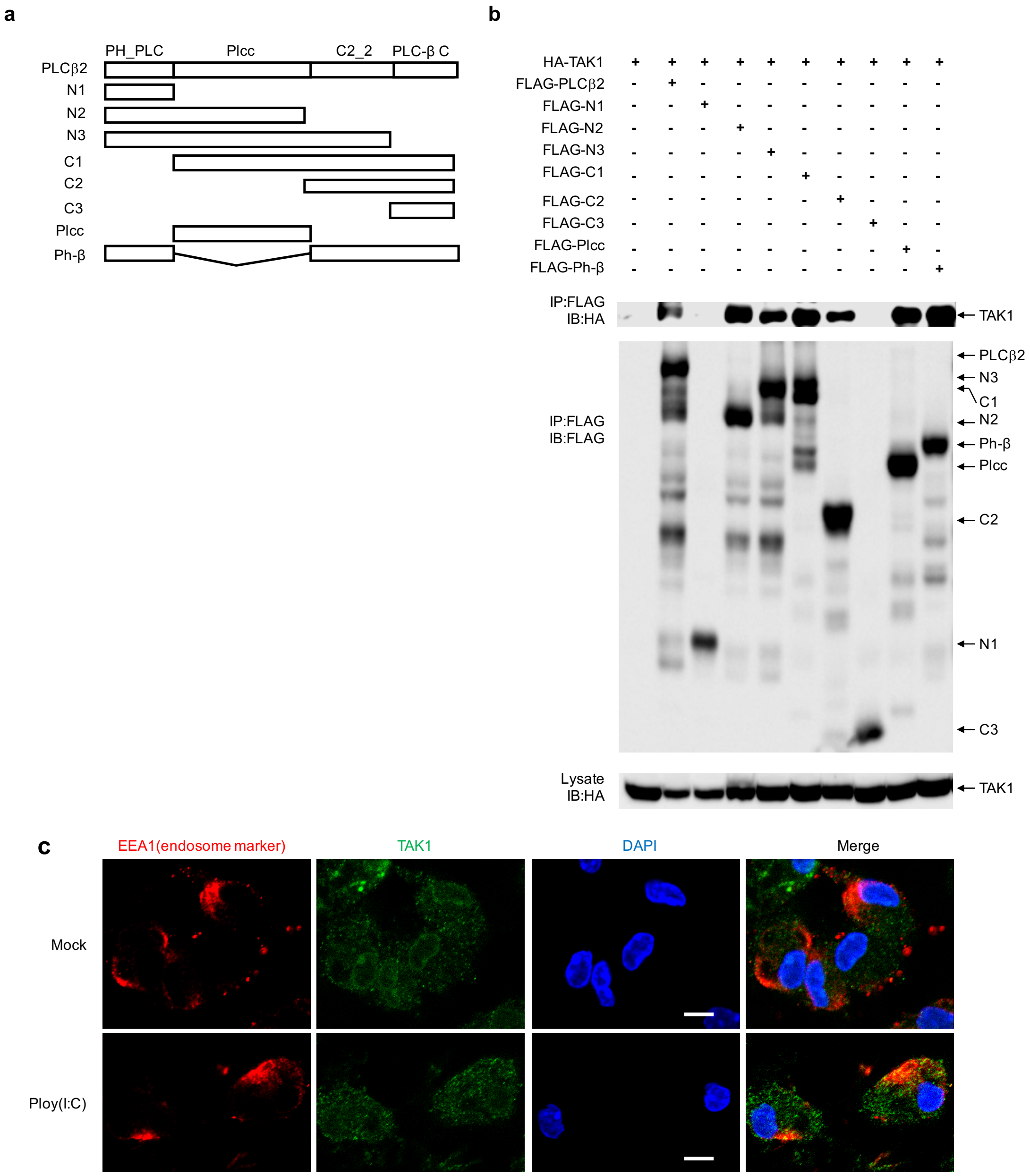

Supplementary Figure 3 PLCβ2 interacts with TAK1 (**a**) Deletion mutants of PLCβ2. (**b**) IP and IB analysis of lysates of HEK293T cell expressing PLCβ2 mutants and TAK1. (**c**) Confocal analysis of location of TAK1 in peritoneal macrophages with mouse anti-TAK1 antibody and EEA1 antibody (endosome marker). Scale bar, 5um. Data are representative of three experiments with at least three independent biological replicates.

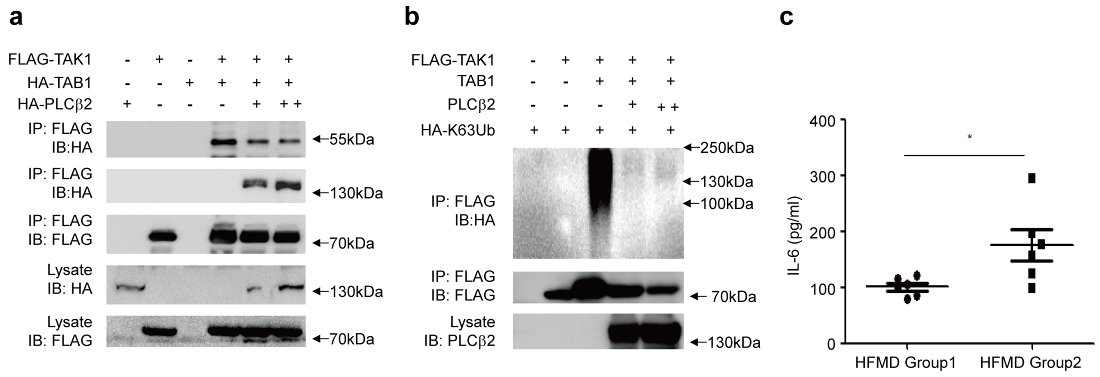

Supplementary Figure 4 PLCβ2 inhibits TAK1 activation (**a**) IB and IP of cell lysates from HEK293 cells expressing TAK1 and TAB1 in the presence or absence of PLCβ2. (**b**) IP and IB analysis of cell lysates from HEK293T cells expressing K63-Ub with TAK1 and TAB1 in the presence or absence of PLCβ2. (**c**) Enzyme-linked immune sorbent assay (ELISA) of IL-6 in serum from HFMD patients. Data are representative of three experiments with at least three independent biological replicates. *$p < 0.05$ by unpaired *t*-test (**c**). Data are representative of three experiments with at least three independent biological replicates.

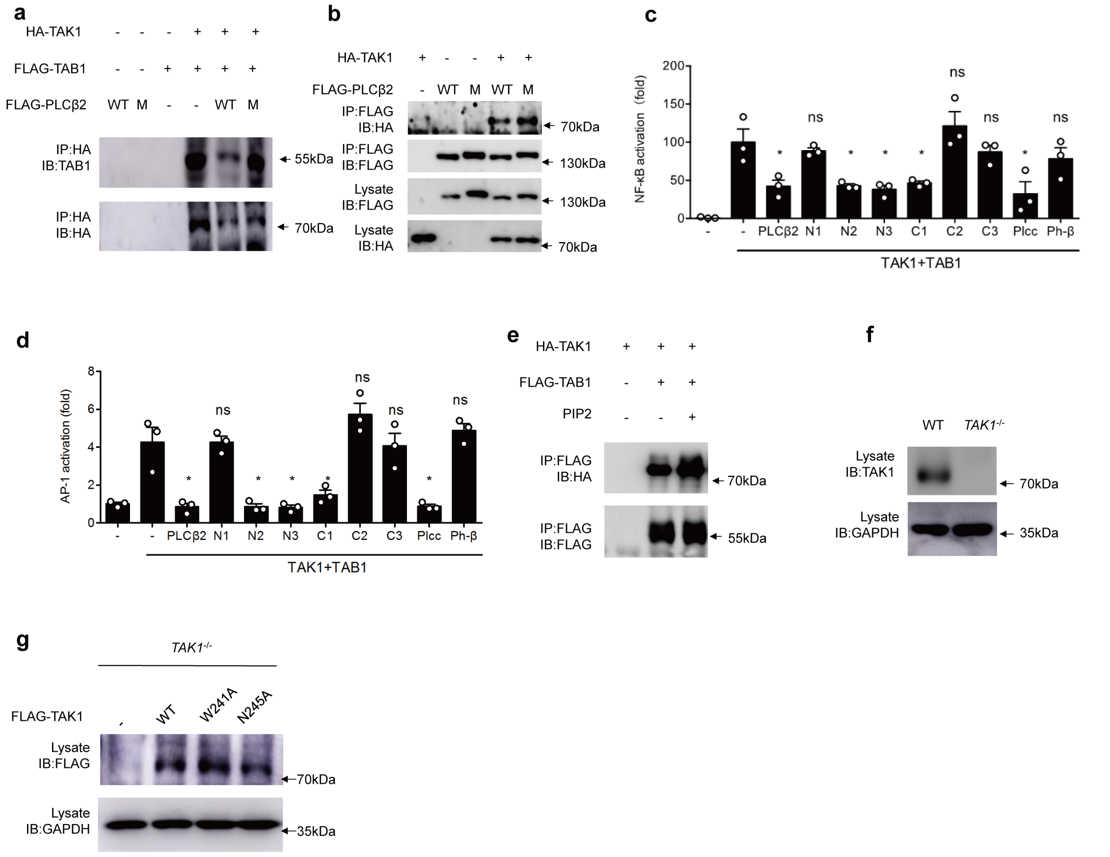

Supplementary Figure 5 PLCβ2 inhibits TAK1 activation via PIP2 (**a**, **b**) IB of cell lysates from HEK293 cells transfected with various plasmids as indicated (**c**, **d**) Reporter assay of NF-κB (**c**) and AP-1 (**d**) activation in HEK293T cells expressing TAK1 and TAB1 in the presence or absence of PLCβ2 or PLCβ2 mutants. (**e**) IB and IP of cell lysates from HEK293 cells expressing HA-tagged TAK1 and FLAG-tagged TAB1 treated with PIP2 in a lipid carrier for 10 mins. (**f**) IB of TAK1 in wild-type (WT) or *TAK1*$^{-/-}$ A549 cells with rabbit anti-TAK1 antibody. (**g**) IB and IP of cell lysates from *TAK1*$^{-/-}$ A549 cells transfected with wild-type TAK1 or TAK1 mutants. *p< 0.05 by unpaired t-test (**c**, **d**). Data are representative of three experiments with at least three independent biological replicates.

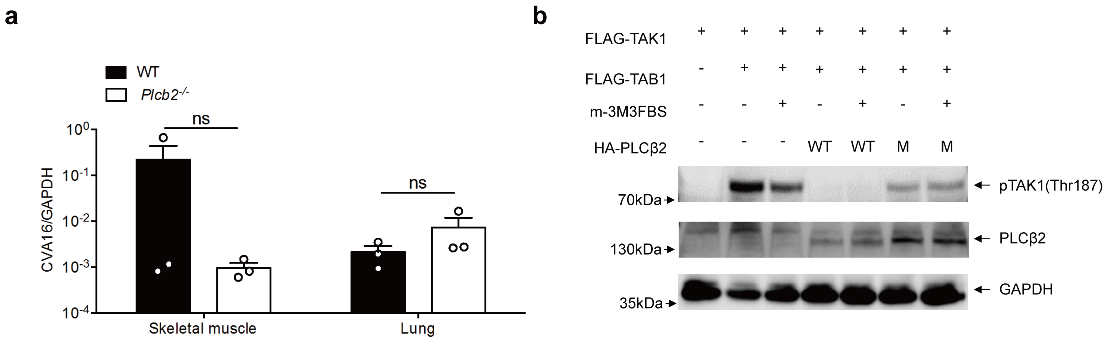

Supplementary Figure 6 PLCB2 PLCβ2-deficient mice are more susceptible to CVA16 infection (**a**) Fourteen-day-old wild-type or *Plcb2*$^{-/-}$ mice were infected intraperitoneally with 50 μl of CVA16 (1.5×10^4 PFU/mouse). The viral loads in the skeletal muscle and lung tissues of infected mice were measured by qRT-PCR on days 3. (**b**) IB and IP of cell lysates from HEK293 cells treated with PLC activator m-3M3FBS for 1h before transfecting plasmid encoding TAK1 and TAB1 in the presence or absence of PLCβ2. Data are representative of three experiments with at least three independent biological replicates. Data are representative of three experiments with at least three independent biological replicates (mean and s.e.m. of n = 3 mice in **a**).

Figure 1d

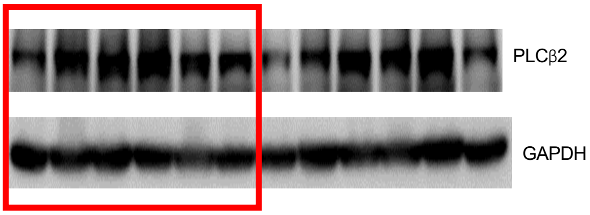

Figure 2f

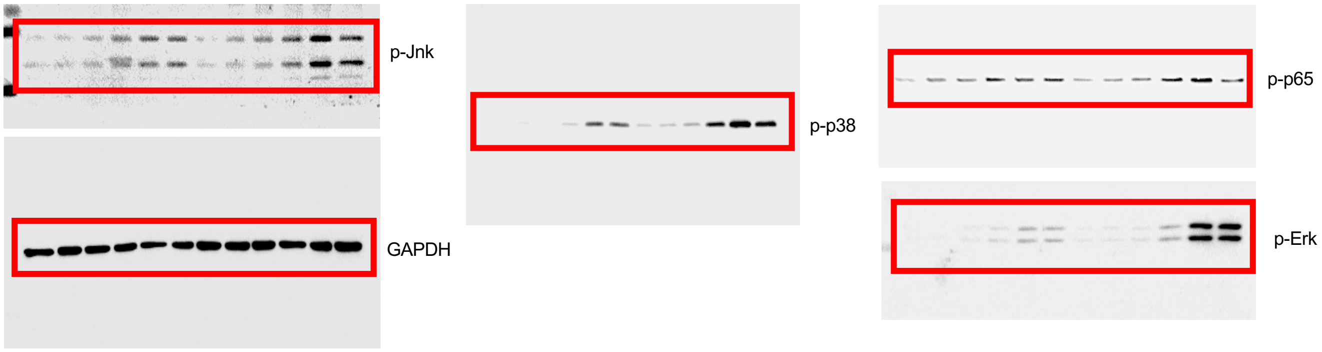

Figure 3a

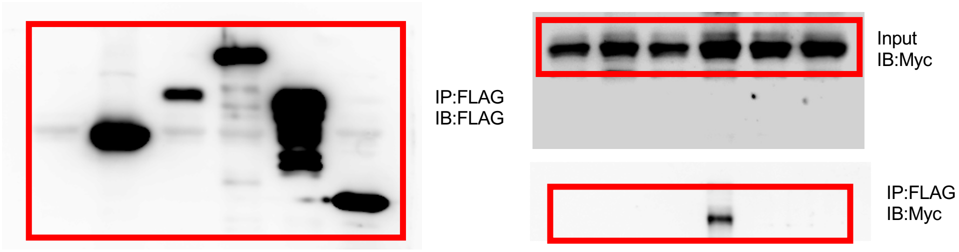

Figure 3c

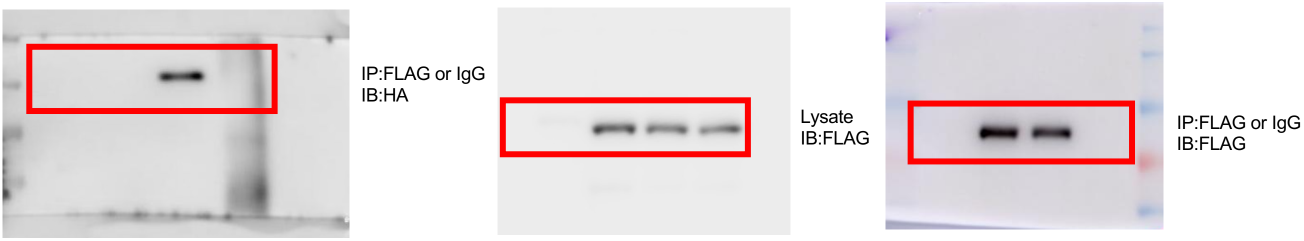

Figure 3d

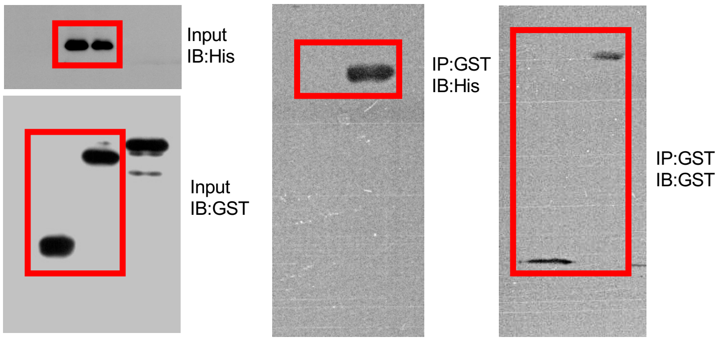

Figure 3e

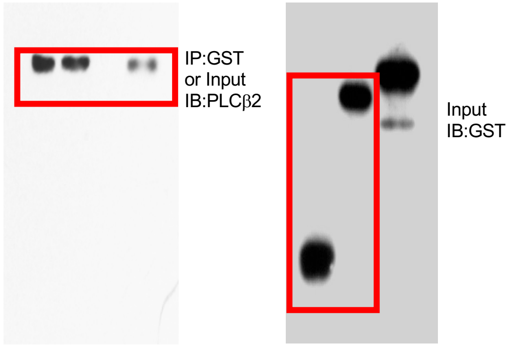

Figure 3h

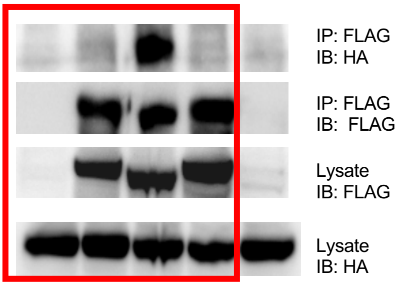

Figure 4d

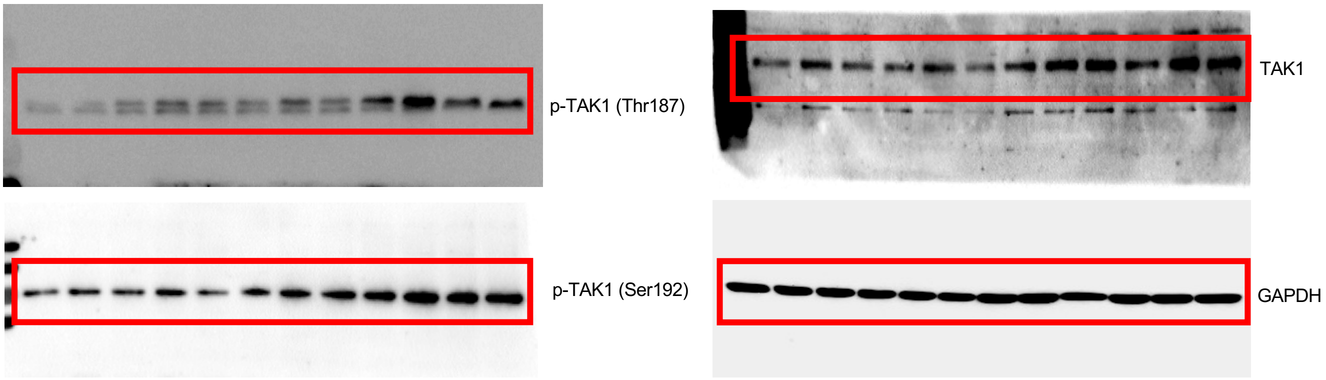

Figure 5c

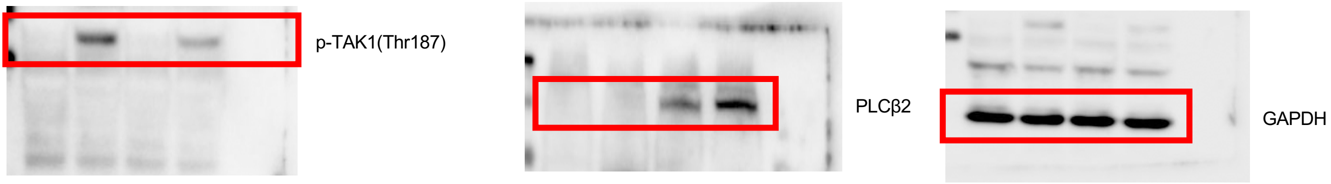

Figure 5d

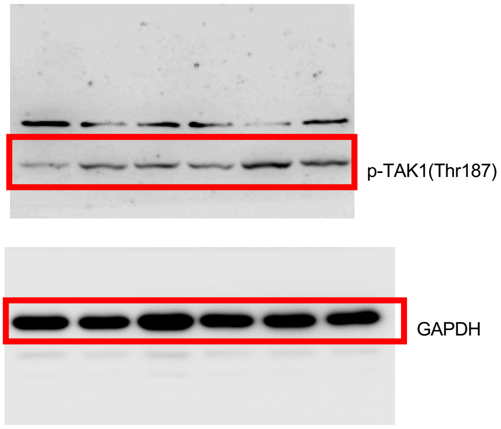

Figure 5e

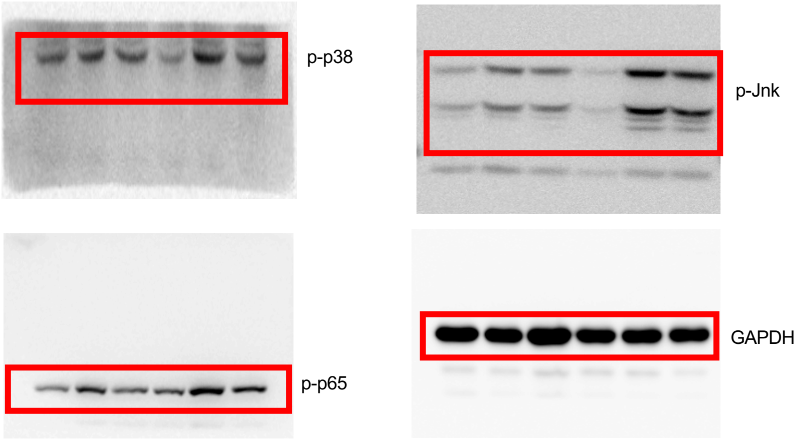

Figure 5f

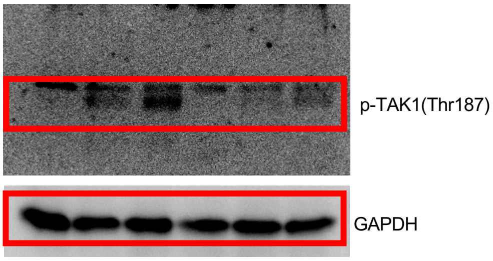

Figure 5g

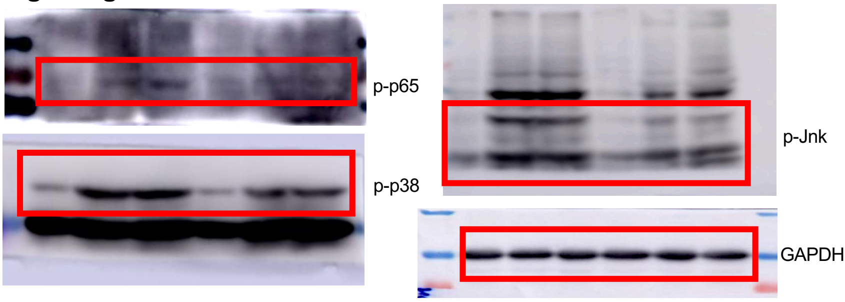

Figure 5j

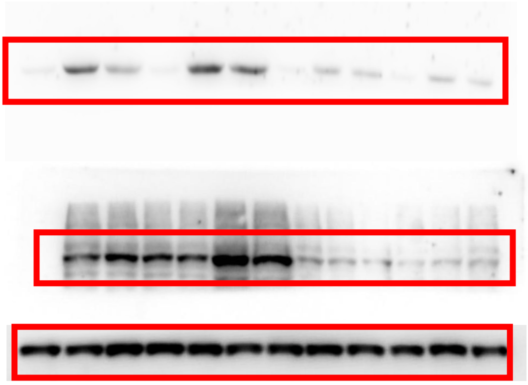

p-p38

p-p65

GAPDH

Figure 5m

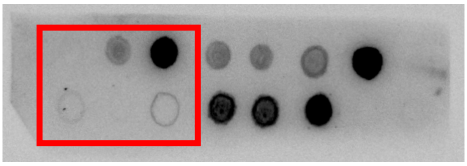

Figure 5n

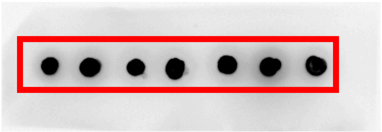

Input:
anti-FLAG

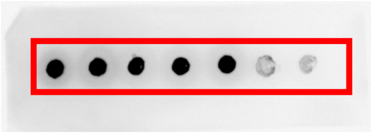

anti-PIP2

Figure 5o

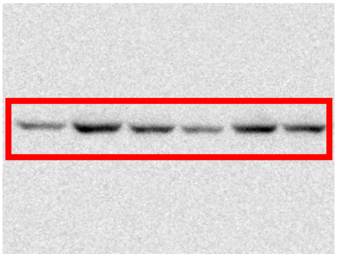

p-p65

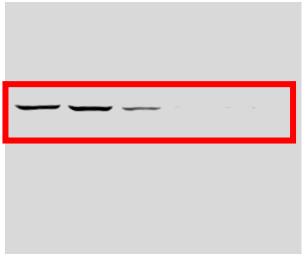

p-TAK1(Thr187)

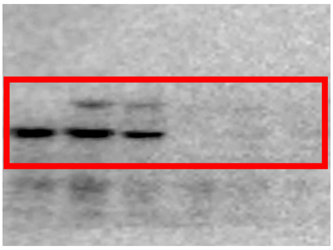

p-Jnk

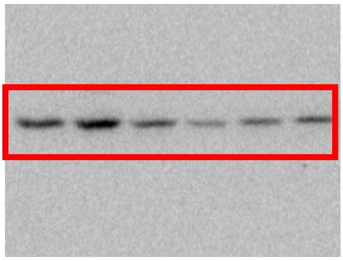

p-p38

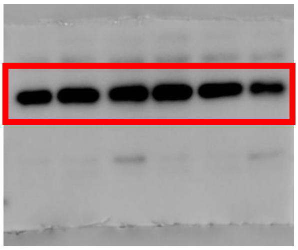

GAPDH

Figure 7a

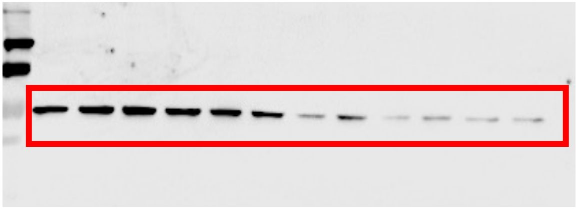

p-TAK1 (Ser192)

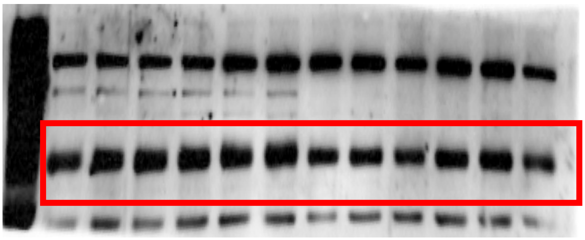

TAK1

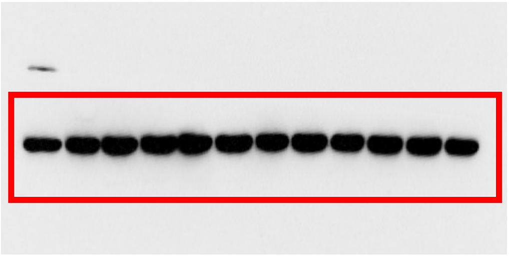

GAPDH

Figure 7b

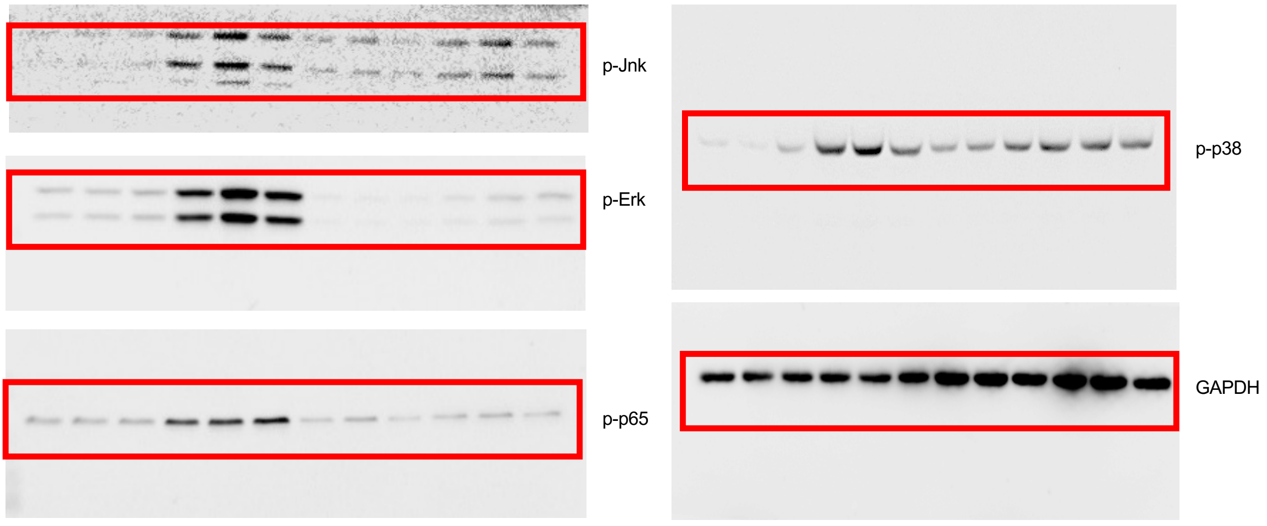

Supplementary Figure 7 Original immunoblots shown in the main Figures
