# Supplementary material for: PLCβ2 negatively regulates the inflammatory response to virus infection by inhibiting phosphoinositide-mediated activation of TAK1
Source: Nat Commun. 2019 Feb 14;10:746. doi: 10.1038/s41467-019-08524-3 (PMC6375925; doi:10.1038/s41467-019-08524-3)
Supplement: Supplementary file 3 — Reporting Summary [file 41467_2019_8524_MOESM3_ESM.pdf]

nature research

Corresponding author(s): Baoxue Ge

Last updated by author(s): Dec 21, 2018

Reporting Summary

Nature Research wishes to improve the reproducibility of the work that we publish. This form provides structure for consistency and transparency in reporting. For further information on Nature Research policies, see Authors & Referees and the Editorial Policy Checklist.

Statistics

For all statistical analyses, confirm that the following items are present in the figure legend, table legend, main text, or Methods section.

n/a	Confirmed	
☐	☒	The exact sample size (*n*) for each experimental group/condition, given as a discrete number and unit of measurement
☐	☒	A statement on whether measurements were taken from distinct samples or whether the same sample was measured repeatedly
☐	☒	The statistical test(s) used AND whether they are one- or two-sided<br>*Only common tests should be described solely by name; describe more complex techniques in the Methods section.*
☒	☐	A description of all covariates tested
☒	☐	A description of any assumptions or corrections, such as tests of normality and adjustment for multiple comparisons
☐	☒	A full description of the statistical parameters including central tendency (e.g. means) or other basic estimates (e.g. regression coefficient) AND variation (e.g. standard deviation) or associated estimates of uncertainty (e.g. confidence intervals)
☐	☒	For null hypothesis testing, the test statistic (e.g. *F*, *t*, *r*) with confidence intervals, effect sizes, degrees of freedom and *P* value noted<br>*Give P values as exact values whenever suitable.*
☒	☐	For Bayesian analysis, information on the choice of priors and Markov chain Monte Carlo settings
☒	☐	For hierarchical and complex designs, identification of the appropriate level for tests and full reporting of outcomes
☒	☐	Estimates of effect sizes (e.g. Cohen's *d*, Pearson's *r*), indicating how they were calculated

Our web collection on statistics for biologists contains articles on many of the points above.

Software and code

Policy information about availability of computer code

Data collection	The confocal data: Leica TCS SP8 confocal laser microscopy system<br>The qRT-PCR data: ViiA 7 Software v1.2.4 on ViiA 7DX<br>The western blot data: ImageQuant LAS 4000mini and Amersham Imager 600
Data analysis	GraphPad Prism 6.0

For manuscripts utilizing custom algorithms or software that are central to the research but not yet described in published literature, software must be made available to editors/reviewers. We strongly encourage code deposition in a community repository (e.g. GitHub). See the Nature Research guidelines for submitting code & software for further information.

Data

Policy information about availability of data

All manuscripts must include a data availability statement. This statement should provide the following information, where applicable:

- Accession codes, unique identifiers, or web links for publicly available datasets
- A list of figures that have associated raw data
- A description of any restrictions on data availability

The authors declare that data supporting the findings of this study are available within the manuscript and Supplementary Information.

Field-specific reporting

Please select the one below that is the best fit for your research. If you are not sure, read the appropriate sections before making your selection.

☒ Life sciences ☐ Behavioural & social sciences ☐ Ecological, evolutionary & environmental sciences

For a reference copy of the document with all sections, see nature.com/documents/nr-reporting-summary-flat.pdf

Life sciences study design

All studies must disclose on these points even when the disclosure is negative.

Sample size	For animal assay, sample sizes were selected empirically from previous experimental experience with similar assays, and/or from sizes generally employed in the field as indicated in the method section (Animal experiments).
Data exclusions	No exclusion of animals were used. For human samples, inclusion criteria was indicated in the method section (Human samples).
Replication	All attempts at replication were successful.
Randomization	No randomization was used. Mice were randomly chosen from 6-8 weeks old female mice as described in Methods section (Animal experiments).
Blinding	Investigators were not blinded to mouse identity. Reported in Methods section (Animal experiments).

Reporting for specific materials, systems and methods

We require information from authors about some types of materials, experimental systems and methods used in many studies. Here, indicate whether each material, system or method listed is relevant to your study. If you are not sure if a list item applies to your research, read the appropriate section before selecting a response.

Materials & experimental systems

n/a	Involved in the study	
☐	☒	Antibodies
☐	☒	Eukaryotic cell lines
☒	☐	Palaeontology
☐	☒	Animals and other organisms
☐	☒	Human research participants
☒	☐	Clinical data

Methods

n/a	Involved in the study	
☒	☐	ChIP-seq
☒	☐	Flow cytometry
☒	☐	MRI-based neuroimaging

Antibodies

Antibodies used	The following antibodies were used: rabbit anti-K63-linkage specific polyubiquitin (5621), rabbit anti-phospho-TAK1 (Thr187) (4536) , rabbit anti-phospho-p65 (3033), rabbit anti-phospho-Erk1/2 (9101), rabbit anti-phospho-p38 (9215), and rabbit anti-phospho-Jnk (9251), all from Cell Signaling Technology; rabbit anti-PLC2 (sc-206), rabbit anti-phospho-TAK1 (Ser192) (sc-130219), rabbit anti-TAK1 (sc-7162) and monoclonal mouse anti-TAK1 (sc-7967) all from Santa Cruz Biotechnology; rabbit anti-TAB1 (ab27983, from Abcam); rabbit anti- EEA1 (PA1-063A, from Invitrogen) monoclonal mouse anti-FLAG M2 affinity gel (A2220), monoclonal mouse anti-HA (H9658), rabbit anti-HA (H6908), rabbit anti-GAPDH (SAB2701826), and rabbit anti-FLAG (F7425), all from Sigma.
Validation	The commercial antibodies are well used and reported in lots of previous publications.

Eukaryotic cell lines

Policy information about cell lines

Cell line source(s)	HEK293T cells (ATCC CRL-3216) obtained from the American Type Culture Collection (ATCC).
Authentication	The cell lines were purchased from ATCC and authenticated by the vendor.
Mycoplasma contamination	All cell lines were routinely tested for mycoplasma contamination. All cell lines used are free of mycoplasma but not described in text.
Commonly misidentified lines (See ICLAC register)	No Commonly misidentified lines were used.

Animals and other organisms

Policy information about studies involving animals; ARRIVE guidelines recommended for reporting animal research

Laboratory animals	Homozygous Plcb2 knockout and Tlr3 knockout mice with C57BL/6 bacground were bred in specific pathogen-free conditions at the Shanghai Laboratory Animal Center. Fourteen-day-old or 6-week-old Plcb2 knockout mice and wild-type control mice were used in the experiments. This was described in Methods section (Mouse strains and Animal experiments parts).
Wild animals	NA
Field-collected samples	NA
Ethics oversight	All animal studies were approved by the Institutional Animal Care and Use Committee of Tongji University as indicated in Method section (Mouse strains part).

Note that full information on the approval of the study protocol must also be provided in the manuscript.

Human research participants

Policy information about studies involving human research participants

Population characteristics	Whole blood samples were obtained from children under 10 years old. Children were clinically diagnosed with HFMD according to the Ministry of Health diagnostic criteria of HFMD (2008), China (http://www.moh.gov.cn/mohbgt/s9503/200812/38494.shtml). Additionally, age-matched healthy children with no history of HFMD were included as controls. This was described in Method section (Human samples part).
Recruitment	Patients were recruited when they came in for hospital.
Ethics oversight	This work received approval from the Clinical Ethics Committee of Xinhua Hospital, which is affiliated with Shanghai Jiaotong University School of Medicine, in accordance with governmental guidelines and institutional policies. Written informed consent was obtained from the next of kin, caretakers, or guardians on behalf of the minors/children participants involved in our study. The ethics committee specifically approved this consent procedure. This was described in Method section (Ethics statement part).

Note that full information on the approval of the study protocol must also be provided in the manuscript.
